# Left brachiocephalic vein aneurysm: a case report

**DOI:** 10.1186/s40792-021-01148-0

**Published:** 2021-03-09

**Authors:** Harushi Ueno, Mari Yazawa, Hideki Tsubouchi, Keita Nakanishi, Tomoshi Sugiyama, Yuka Kadomatsu, Masaki Goto, Naoki Ozeki, Shota Nakamura, Takayuki Fukui, Masato Mutsuga, Toyofumi Fengshi Chen Yoshikawa

**Affiliations:** 1 Department of Thoracic Surgery, Nagoya University Graduate School of Medicine, 65 Tsurumai-cho, Showa-ku, Nagoya, 466-8550 Japan; 2 Department of Cardiac Surgery, Nagoya University Graduate School of Medicine, 65 Tsurumai-cho, Showa-ku, Nagoya, 466-8550 Japan

**Keywords:** Left brachiocephalic vein, Innominate vein, Venous aneurysm

## Abstract

**Background:**

Aneurysm of the left brachiocephalic vein is a very rare clinical disease and only 40 cases have been reported so far.

**Case presentation:**

The patient was a 61-year-old woman with no related medical history. She underwent CT to investigate the cause of a cough and a mass was noted in the anterior mediastinum. Dynamic computed tomography with contrast medium injected into the left basilic vein demonstrated the venous aneurysm with blood flow to the left brachiocephalic vein. The patient had no symptoms, but because of the risk of pulmonary infarction and aneurysm rupture, the aneurysm was surgically resected. A median sternotomy was a reasonable approach because of the fragility of the venous aneurysm wall with little working space in the anterior mediastinum.

**Conclusions:**

We diagnosed an aneurysm of the left brachiocephalic vein on preoperative imaging and excised it through a median sternotomy. The venous wall was thin and fragile in some areas and so this approach was appropriate in view of the possibility of intraoperative injury.

## Background

Aneurysms of the left brachiocephalic vein (LBCV) are extremely rare; only 40 cases have been reported so far. They are usually asymptomatic and most often discovered incidentally [1].

Recent reports have indicated that surgery is the treatment in many cases because of the risk of thrombus formation and rupture of the aneurysm, but nearly half the patients are followed up and no treatment guidelines exist [2]. We report a case of LBCV aneurysm without internal thrombus that was diagnosed on contrast medium-enhanced computed tomography (CT) of the chest and removed via median sternotomy.

## Case presentation

**Fig. 1:**
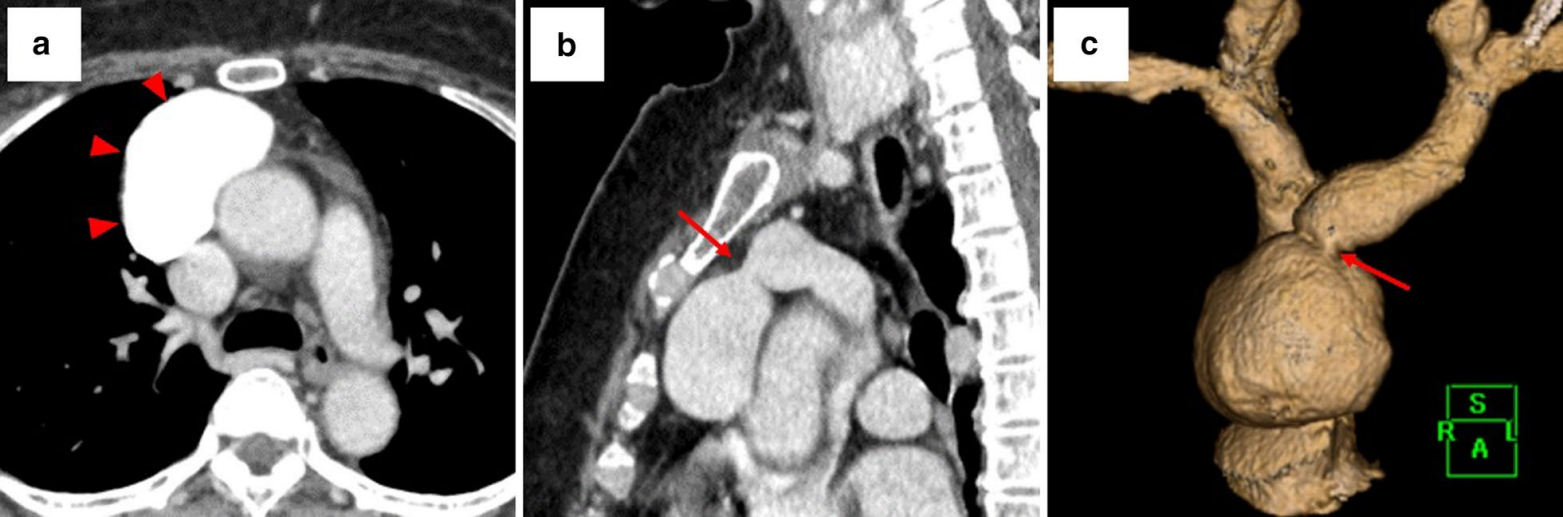
The inside of the aneurysm showed uniform uptake of contrast material and there were no areas of poor contrast that were suggestive of thrombosis (**a**). The aneurysm showed the same absorption value as the brachiocephalic veins, and its neck was 8 mm in diameter (**b**, **c**)

**Fig. 2:**
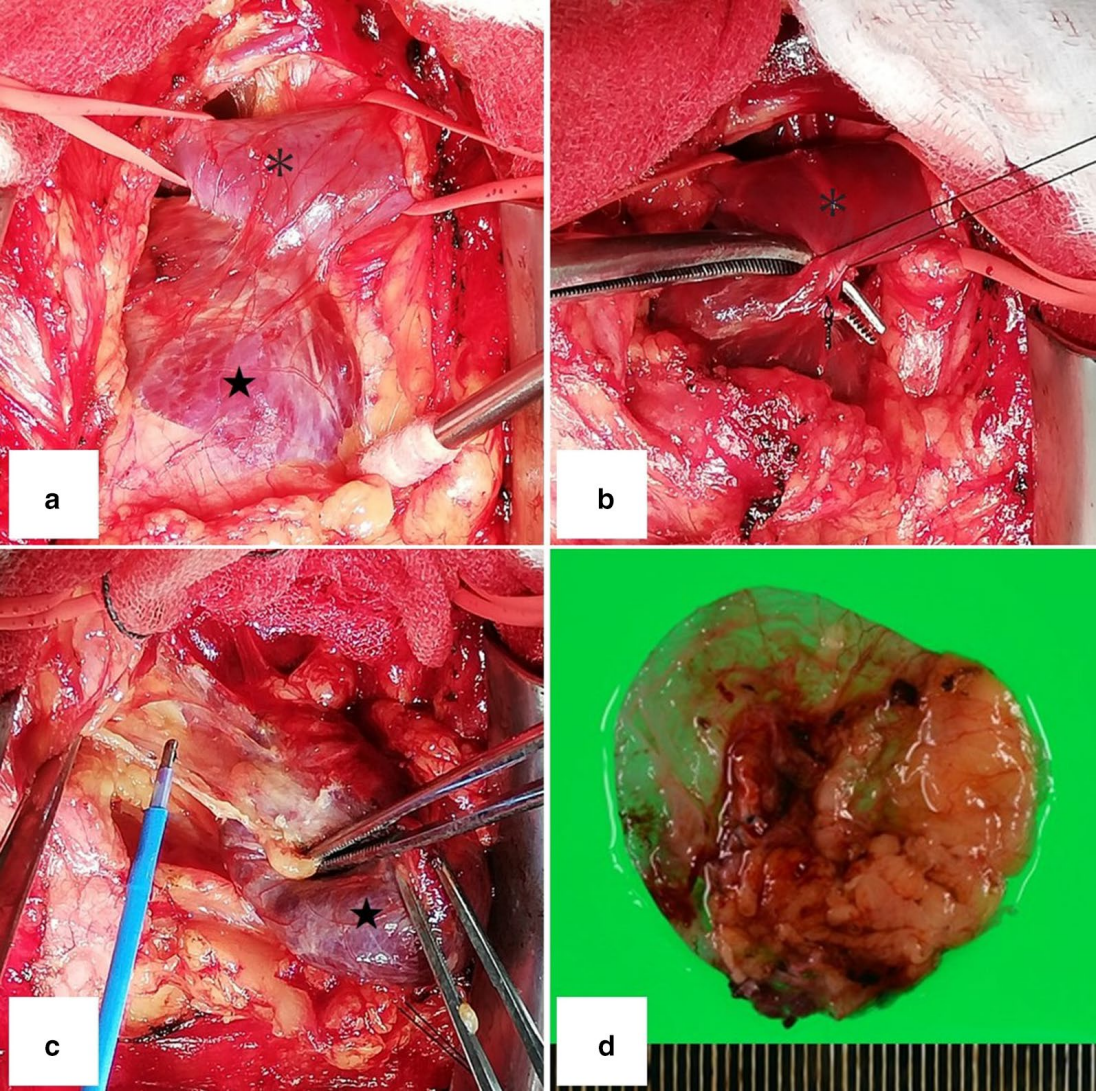
The aneurysm (star) was easily dissected from the thymic adipose tissue for the most part, but parts of the aneurysm wall were fragile. The central and peripheral sides of the brachiocephalic vein (asterisk) were secured (**a**). The brachiocephalic vein (asterisk) and aneurysm (star) were attached by the neck of the aneurysm, which was 8 mm in diameter and was ligated and dissected (**b**). Aneurysm (star) was not connected to superior vena cava (**c**). Some parts of the venous walls were thin and fragile (**d**)

The patient was a 61-year-old woman with no related medical history. She underwent CT to investigate the cause of a cough and a mass was noted in the anterior mediastinum. Blood biochemistry tests including tests for tumor markers yielded normal results. Dynamic CT of the chest was performed with contrast medium (Iopamiron 100 ml Inj.Syringe; Bayer Yakuhin, Ltd, Osaka, Japan), which was injected via a 370 syringe into the left basilic vein. The mass measured 54 $\times$ 47 $\times$ 31 mm and its neck was 8 mm in diameter; its boundaries were well defined, and it was cystic and continuous with the inferior margin of the LBCV. In the pulmonary artery phase of scanning, the CT value of the tumor was comparable with that of the LBCV, and in the equilibrium phase, no contrast deficit indicated a thrombus (Fig. 1a–c). We used Synapse Vincent (v. 4.3) (Fujifilm) software to reconstruct vessel image by CT. On this basis, we diagnosed LBCV aneurysm. The aneurysm was resected via median sternotomy. A mass containing dark brown content was identified within the thymic adipose tissue (Fig. 2a). By dissecting the LBCV, we identified the neck of the aneurysm. After the peripheral and central sides of the LBCV were secured, the neck of the aneurysm was excised by double ligation (Fig. 2b). For the most part, dissection from the thymic adipose tissue was easy and there was no connection between LBCV aneurysm and superior vena cava (Fig. 2c). Some bleeding occurred in areas with thin venous walls, which were repaired by suture. The operative time was 87 min and blood loss was 120 mL. Parts of the wall of the aneurysm were thin (Fig. 2d). Pathological findings confirmed a diagnosis of venous aneurysm with dilated lumen. No pyogenic inflammation or malignant neoplasm was found. The patient’s postoperative course and follow-up monitoring were uneventful.

## Conclusions

LBCV aneurysm is a rare condition for which no treatment guidelines exist. Congenital conditions, trauma, inflammation, vascular regression changes, excessive blood flow pressure, and malignancy have been noted as causes of LBCV aneurysm. Many cases of LBCV aneurysm are asymptomatic and diagnosed incidentally. In some cases, a close examination of pain, cough, hoarseness, and respiratory distress may lead to diagnosis [2].

When the contrast agent is administered into the left median cubital vein, the distance to the cardiac cavity is normally long, which may delay the arrival time of the contrast agent, interfere with arterial delineation, and prevent sufficient contrast agent from reaching the target vessel. Therefore, the contrast agent is usually injected into the right basilic vein. However, delivery through the right basilic vein may not enable sufficient contrast agent to reach the LBCV, which may prevent visualization. Moreover, if extensive thrombus occupies the venous aneurysm, the flow of contrast agent may be blocked and the mass may not be confirmed by imaging [3]. We injected the contrast agent into the left basilic vein because we suspected venous aneurysm and we produced a perfect image that enabled us to make a preoperative diagnosis.

Surgery is an option when compression of an organ by the mass causes symptoms, bleeding in the anterior mediastinum results from rupture of the mass, or thrombus formation increases the risk of pulmonary infarction. However, many asymptomatic and untreated cases have been reported and the decision to treat them is controversial. Shen et al. reported a case of thrombus in an aneurysm that was noted on preoperative imaging [4], and thrombi have been reported in 8 of 24 cases of saccular aneurysms of the LBCV [2, 5–8]. Although no rupture of LBCV aneurysms has been reported, the risk of rupture is probably proportional to the size of the mass, as with aortic aneurysms, although it is a low-pressure system. Lohrenz et al. reported a case in which the aneurysm increased in size during follow-up [9]. On the basis of these reports, we decided to resect the aneurysm, although we found no thrombus in our patient’s aneurysm.

In view of the risk of thrombus formation and dissemination within an aneurysm, it is necessary to initially block blood flow between the aneurysm and brachiocephalic veins. Because the space between the sternum and the aortic root is narrow, the aneurysm wall can become compressed and can rupture during thoracoscopic surgery. In our patient, the neck was 8 mm in diameter and the ligation process was considered easy, but median sternotomy was the approach chosen because of the difficulty in securing the working space. The decision to excise an aneurysm should take into account the condition of the neck and the size of the aneurysm. In many cases, the maximum diameter of the aneurysm hampers the safety of the working space and median sternotomy might be preferable.

In conclusion, we diagnosed an LBCV aneurysm on preoperative imaging and excised it through a median sternotomy. The venous wall was thin and fragile in some areas and so this approach was appropriate in view of the possibility of intraoperative injury.

## Data Availability

The data that support the findings of this study are available from the corresponding author upon reasonable request.

## Abbreviations

LBCV: Left brachiocephalic vein
CT: Computed tomography
